# Validation and reliability of the Dutch version of the EORTC QLQ-NMIBC24 Questionnaire Module for patients with non-muscle-invasive bladder cancer

**DOI:** 10.1186/s41687-021-00372-4

**Published:** 2021-09-20

**Authors:** Theodora M. Ripping, Ellen Westhoff, Neil K. Aaronson, Mieke Van Hemelrijck, Elke Rammant, J. Alfred Witjes, Lambertus. A. Kiemeney, Katja K. H. Aben, Alina Vrieling

**Affiliations:** 1 Netherlands Comprehensive Cancer Organisation, Utrecht, The Netherlands; 2 Department for Health Evidence, Radboud Institute for Health Sciences, Radboud University Medical Center, PO Box 9101, 6500 HB Nijmegen, The Netherlands; 3 Division of Psychosocial Research and Epidemiology, The Netherlands Cancer Institute, Amsterdam, The Netherlands; 4 Faculty of Life Sciences and Medicine, Translational Oncology and Urology Research (TOUR), King’s College London, London, UK; 5 Department of Human Structure and Repair, Ghent University, Ghent, Belgium; 6 Department of Urology, Radboud Institute for Molecular Life Sciences, Radboud University Medical Center, Nijmegen, The Netherlands

**Keywords:** Bladder cancer, Quality of life, Validation studies, EORTC questionnaire

## Abstract

**Background:**

The European Organisation for Research and Treatment of Cancer (EORTC) quality of life questionnaire for non-muscle invasive bladder cancer (QLQ-NMIBC24) has been available and applied for some years now, but has yet to undergo a full comprehensive psychometric evaluation. The aim of this study was to investigate the psychometric properties of the Dutch version of the EORTC QLQ-NMIBC24 questionnaire in patients with low, intermediate and high risk NMIBC.

**Methods:**

We included patients newly diagnosed with NMIBC participating in the multicenter, population-based prospective cohort studies UroLife or BlaZIB. Psychometric evaluation included examination of the structural validity, reliability (i.e. internal consistency and test–retest reliability), construct validity (i.e. divergent validity and known-groups validity), responsiveness and interpretability.

**Results:**

A total of 1463 patients who completed the baseline questionnaire of UroLife (n = 541, response rate 50%) or BlaZIB (n = 922, response rate 58%) were included. The percentage of missing responses were low for all non-sex related scales (< 1%) and ranged between 6.9% to 50.0% for sex-related scales. More than 15% of the patients obtained the lowest possible scores on nearly each scale (floor effect). The structural validity was adequate; the confirmatory factor analysis showed satisfactory results and all items of multiple items scales had higher within- than between-scale correlations. Reliability of the questionnaire was adequate for most multiple item scales (Cronbach’s α ≥ 0.70 and intraclass correlation coefficient ≥ 0.70), with exception of the scales ‘malaise’ and ‘bloating and flatulence’. The questionnaire also showed good construct validity; it showed low correlations with the items of the EORTC core questionnaire and was able to measure differences between risk-based subgroups. The responsiveness of the questionnaire was good, but the interpretability, i.e. minimal important change, could not be determined.

**Conclusions:**

This study shows that the measurement properties of the EORTC QLQL-NMIBC24 are good; it has a good structural validity, reliability (i.e. internal consistency and test–retest reliability), construct validity (i.e. divergent validity and known-group validity), and responsiveness. Interpretability could not be assessed. This questionnaire can be used to measure and monitor health-related quality of life of patients with NMIBC.

**Supplementary Information:**

The online version contains supplementary material available at 10.1186/s41687-021-00372-4.

## Background

The majority (75%) of new bladder cancer patients are diagnosed with non-muscle invasive bladder cancer (NMIBC) and undergo a transurethral resection (TURBT) [1]. Dependent on the tumour’s risk profile, this is followed by a single chemotherapy instillation (low-risk tumours), adjuvant intravesical chemotherapy or Bacillus Calmette Guerin (BCG) for a maximum of 1 year (intermediate-risk tumours), or BCG maintenance for 1–3 years (high-risk tumours) [1]. As both the disease and its treatment can affect functional health and symptom experience, the European Organisation for Research and Treatment of Cancer (EORTC) developed a health-related quality of life (HRQoL) questionnaire specifically for patients with NMIBC, the EORTC Quality of Life Questionnaire (QLQ)-NMIBC24 [2, 3]. This questionnaire module was designed to complement the EORTC core HRQoL questionnaire, the QLQ-C30. The QLQ-NMIBC24 is already partially validated (i.e. content validity) but still needs to undergo psychometric testing in a large international group of patients (phase III validated EORTC module) [4]. To date, three studies have investigated the psychometric properties of the QLQ-NMIBC24 questionnaire showing its psychometric robustness [2, 5, 6]. One study examined and revised the scale structure and evaluated the internal consistency, known group validity, and responsiveness of the questionnaire in a British patient population [2]. The other two studies evaluated the psychometric properties of the Danish and Korean translation of the questionnaire, respectively [5, 6]. However, no full comprehensive evaluation of the psychometric properties of the QLQ-NMIBC24, which is required to judge the appropriateness of the measure, has been performed. Test–retest reliability, interpretability of change scores [7] and the performance of the NMIBC24 has not yet been evaluated in different risk groups of NMIBC patients or among Dutch-speaking patients.

Therefore, the aim of this study was to examine the structural validity, reliability (i.e. internal consistency and test–retest reliability), construct validity (i.e. divergent validity and known group validity), responsiveness and interpretability of the Dutch version of the QLQ-NMIBC24 [3] in patients with low, intermediate and high risk NMIBC.

## Methods

### Study design and participants

Dutch bladder cancer patients participating in the UroLife (Urothelial cell cancer: Lifestyle, prognosis and quality of Life) or BlaZIB (‘BlaaskankerZorg In Beeld’, clinical trial number: NL8106) studies were included in the current analysis. Both studies are population-based, multicenter prospective cohort studies recruiting newly diagnosed bladder cancer patients based on notifications from the nationwide network and registry of histopathology and cytopathology in the Netherlands (PALGA) and successive registration in the Netherlands Cancer Registry (NCR). The main aim of the Urolife study is to evaluate the association between lifestyle habits and the risk of recurrence and progression and HRQoL of patients with NMIBC. BlaZIB aims to gain insight in bladder cancer care and to identify barriers and modulators for optimal care. More detailed information on these studies can be found elsewhere [8, 9]. For this analysis, patients diagnosed with NMIBC (stage Ta, T1, Tis) between April 1, 2014 and March 18, 2016 were selected from the UroLife study, and patients diagnosed with high-risk NMIBC (stage T1 and Tis) between November 1, 2017 and July 7, 2019 were selected from the BlaZIB study. All patients were Dutch speaking, between 18 and 80 years old, and treated with a transurethral resection. This study was performed in line with the principles of the Declaration of Helsinki. The Committee for Human Research in the region Arnhem-Nijmegen provided ethical approval for the UroLife study (CMO 2013-494) and deemed the BlaZIB study exempt from ethical review under the Medical Research Involving Human Subjects Act (WMO). Both studies were approved by the ethical review board of the NCR. Written informed consent was obtained from all patients participating in UroLife or BlaZIB.

### Data collection

**Fig. 1:**
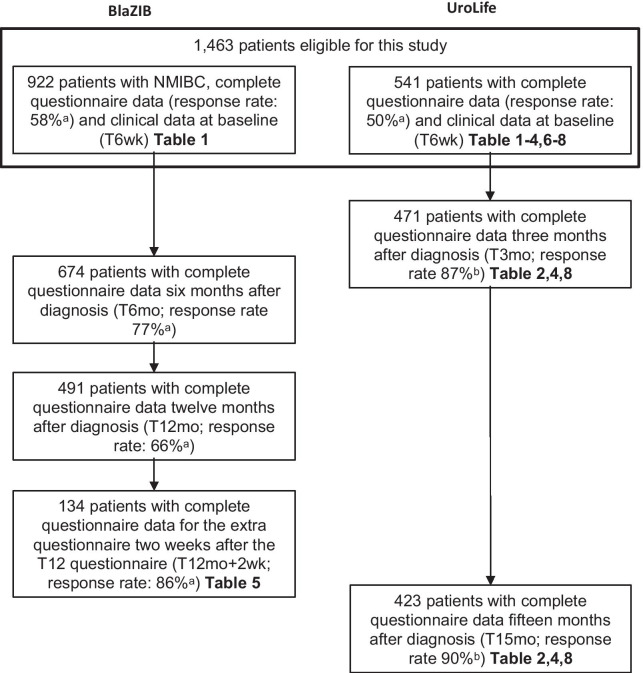
Flowchart BlaZIB and Urolife. *NMIBC* non-muscle invasive bladder cancer. ^a^Percentage of patients that completed the questionnaire after being invited to fill in the questionnaire. ^b^Percentage of patients that completed the questionnaire after completing the previous questionnaire

Both studies collected self-reported questionnaire data online or on paper 6 weeks after diagnosis (T6wk). The online questionnaires were collected via the data collection tool of the Patient Reported Outcomes Following Initial treatment and Long term Evaluation of Survivorship (PROFILES) registry [10]. Baseline data (T6wk) and follow-up data collected at 3 months (T3mo) and 15 months (T15mo) after diagnosis in the UroLife study, and at 6 months (T6mo) and 12 months (T12mo) after diagnosis in the BlaZIB study were used for the current analysis. The measurement points of UroLife were based on the treatment regimen of patients diagnosed with NMIBC, i.e. shortly after histological confirmation of the tumour (T6wk), at time of cystoscopy to investigate whether the tumour was successfully removed (T3mo), and long-term follow-up (T15mo). For the BlaZIB study, including also patients with Muscle Invasive Bladder Cancer, different measurement points were selected. Because of the nonconforming measurement points, most analyses were based on UroLife data and only supplemented with BlaZIB data where necessary (i.e. test–retest, interpretability of change scores; see also Fig. 1). The baseline questionnaires assessed demographics, smoking history, and comorbidity. HRQoL was assessed at T6wk and during follow-up using the EORTC-QLQ-C30 and the QLQ-NMIBC24 questionnaires [2, 11]. Patients who underwent a cystectomy were not or no longer invited to participate in the UroLife study.

In order to assess the test–retest reliability and standard error of measurement (SEM), patients who completed the BlaZIB T12mo questionnaire between March 1st 2019 and December 7th 2019 received an additional questionnaire 2 weeks after the T12mo questionnaire (T12mo + 2wk). In total, 134 patients diagnosed with NMIBC completed the T12mo + 2wk questionnaire (response rate 86.5%). This questionnaire included the QLQ-NMIBC24 and four additional questions to assess whether the symptoms – in terms of urinary, bowel, sexual and total function – had decreased, remained the same or increased compared to the T12mo questionnaire (three-point Likert scale, see Additional file 1: Appendix A). Patients whose symptoms remained the same were regarded as stable and included in the test–retest analysis.

In order to assess the minimal important change (MIC), the follow-up questionnaires of BlaZIB (T6mo, T12mo) contained an anchor question to assess changes over time., i.e. ‘Did your bladder cancer-specific complaints (urinary, bowel, sexual function and overall) change compared to your complaints at diagnosis?’. Patients were asked to score their change on a nine-point Likert scale ranging from 1 (worse than ever) to 9 (no complaints anymore) for urinary, bowel, sexual and total function, separately [12]. We clustered the answers into three categories: importantly deteriorated (1–3), not importantly changed (4–6) and importantly improved (7–9) [13].

### HRQoL questionnaires

The EORTC QLQ-C30 is the core HRQoL questionnaire of the EORTC and measures the HRQoL of cancer patients. The questionnaire consists of 30 items organized into a global health status scale, five functioning scales (physical, role, cognitive, emotional, and social), three symptom scales (fatigue, pain, and nausea and vomiting) and six single items (dyspnoea, insomnia, loss of appetite, constipation, diarrhea, and financial impact) [11].

The QLQ-NMIBC24 is an EORTC module for patients diagnosed with NMIBC and should be administered in addition to the core questionnaire (EORTC-QLQ-C30) [4]. The module includes constructs specific to the tumour site and treatment of NMIBC. The QLQ-NMIBC consists of 24 items organized into six scales (urinary symptoms, malaise, future worries, bloating and flatulence, sexual functioning, and male sexual problems) and five single items (intravesical treatment issues, sexual intimacy, risk of contaminating partner, sexual enjoyment, female sexual problems) [3].

All items were scored on a four-point Likert scale ranging from 1 (not at all) to 4 (very much), with the exception of the global health status items, which employ a seven-point scale ranging from 1 (very poor) to 7 (excellent). Scores of items were summed and linearly transformed to 0–100 scales and missing data were imputed according to the EORTC guideline [14]. Higher scores on functioning scales and global health status represent better functioning, while higher scores on the symptom scales indicate more symptom burden. Higher scores on the scales and items of the QLQ-NMIBC24 should be interpreted as more symptom burden, with exception of the sexual function scale and sexual enjoyment where higher scores represent better functioning.

### Statistical analysis

Floor and ceiling effects were examined for each scale at each assessment point. If more than 15% of the patients scored at the lowest or highest end of the scale, the scale was considered to have a floor or ceiling effect, respectively [15]. Multitrait scaling analysis and Confirmatory factor analysis (CFA) were performed to validate the constructs of the QLQ-NMIBC24. Convergent validity was defined as a correlation of 0.40 or greater between an item and its own scale. Discriminant validity was defined a as correlation of less than 0.40 between an item and any other scale [2, 16]. Maximum Likelihood (ML) was used as estimator in the CFA and missing items were imputed using Full Info Max Likelihood (fiml). Model-data-fit of the CFA was assessed with model chi-square, the Comparative Fit Index (CFI), Root Mean Square Error of Approximation (RMSEA) and Standardized Root Mean Square Residual (SRMR). Model chi-square > 0.05, CFI ≥ 0.95, RMSEA < 0.05 and SRMR < 0.05 indicate a good fit, and CFI > 0.90 and both RMSEA and SRMR > 0.05 but < 0.08 indicate an acceptable fit [15, 17]. Internal consistency was assessed with Cronbach’s α. A Cronbach’s α of 0.70 or higher was considered adequate for group level comparisons. Test–retest reliability was assessed based on the questionnaires administered at T12mo and T12mo + 2wk using the intraclass correlation coefficient for absolute agreement (ICC; two-way mixed model, single measure) [18]. An ICC value of 0.70 or higher was considered acceptable.

Divergent validity of the QLQ-NMIBC24 was assessed by calculating the Spearman correlation coefficients between the scales of the QLQ-C30 and QLQ-NMIBC24 [19]. Based on previous studies, we expected in general low to moderate correlations (< 0.40) between the scales of both questionnaires. Previous studies have shown that malaise was moderately to strongly correlated (> 0.40) with all the scales of the QLQ-C30 [2, 6, 16]. The urinary symptom scale has previously also shown to be moderately (0.40–0.69) correlated with role function, cognitive function, social function, fatigue, nausea and vomiting, and pain [2, 6]. At last, future worries was expected to be moderately correlated to the emotional function scale of the QLQ-C30 [2] and fatigue [6].

Known group validity was assessed by comparing patients with low, intermediate and high risk NMIBC using independent t-tests. Patients were divided into risk groups based on the European Association of Urology (EAU) guidelines [1] without taking into account the tumour size (not available) and the recurrent nature of the tumour (only primary tumours included). We hypothesized that patients with high risk NMIBC would have more urinary symptoms, malaise, future worries and intravesical treatment issues at T6wk than patients with low risk NMIBC.

Responsiveness to change was examined using all three questionnaires of the UroLife study (i.e. T6w, T3mo and T15mo) using paired t-tests. We hypothesized that differences on the scales of the NMIBC24 would only be small between T6wk and T3mo, but that symptoms and complaints decrease from T6wk to T15mo. Effect sizes (ESs) were calculated using Cohen’s d statistic (mean difference divided by pooled standard deviation). These provide a distribution-based estimate of the magnitude of mean differences/changes, where an ES of 0.2 is considered small, 0.5 moderate, and 0.8 large [20].

MIC was assessed using the visual-anchor distribution method of *De Vet *et al. [13]. This method determines the smallest change in scores of the QLQ-NMIBC24 that are regarded as either improvement or deterioration by taking into account the variability and importance of the scores. To determine the importance of the scores, an external anchor is used. Correlations between the anchor-question and the scales of the QLQ-NMIBC24 were assessed to determine the adequacy of the anchor (r ≥ 0.40) (i.e. does the anchor question measures the same as the change scores?). Then, patients were subdivided into three groups (importantly deteriorated, not importantly changed and importantly improved) using the anchor question and for each group the distribution of the changes scores was plotted. The optimal receiver operating curve (ROC) was considered to be the MIC value.

The CFA was conducted with the software package R using the “lavaan” package [21]. ICCs were calculated in STATA version 16.0 (StataCorp LLC, College Station, Texas, USA) and SEMs were calculated in SAS (SAS Institute, Cary, North Carolina, USA). All other statistical analyses were executed using SPSS version 25 (IBM Corporation, Armonk, New York, USA). P values < 0.05 were considered statistically significant.

## Results

### Patient characteristics and data quality

Fifty percent of the NMIBC patients invited for UroLife and 58% of the NMIBC patients invited for BlaZIB completed the baseline questionnaire, resulting in a total number of 1463 eligible patients for this study (Fig. 1). Figure 1 presents the number of completed questionnaires and the response rates at follow-up. The majority of the patients were male (81%) (Table 1). Patients participating in UroLife were, on average, younger (66 vs. 72 years), more often female (21% vs. 17%), living together with a partner (85% vs. 77%) and employed (42% vs. 24%) than those participating in BlaZIB.

**Table 1:** Sociodemographic characteristics of the NMIBC patients

	All patients (n = 1463)		BlaZIB		Urolife							
			High-risk (n = 922)		All (n = 541)		Low risk (n = 99)		Intermediate risk (n = 250)		High risk (n = 192)	
n (%)	N	(%)	N	(%)	N	(%)	N	(%)	N	(%)	N	(%)
*Patient characteristics*												
Age (years), mean (SD)	70	(10)	72	(10)	66	(9)	65	(10)	66	(9)	67	(7)
Gender (% male)	1187	(81%)	761	(83%)	426	(79%)	68	(69%)	196	(78%)	162	(84%)
Living situation												
With partner	1165	(80%)	707	(77%)	458	(85%)	79	(80%)	212	(85%)	167	(87%)
Without partner	298	(20%)	215	(23%)	83	(15%)	20	(20%)	38	(15%)	25	(13%)
Employment												
Paid job	349	(24%)	171	(19%)	175	(32%)	31	(31%)	89	(36%)	55	(29%)
No paid job	104	(7%)	49	(5%)	55	(10%)	13	(13%)	25	(10%)	17	(9%)
Retired	1010	(69%)	699	(76%)	311	(58%)	55	(56%)	136	(54%)	120	(62%)
Comorbidity^a^												
0	299	(20%)	212	(23%)	87	(16%)	12	(12%)	41	(16%)	34	(18%)
1	406	(28%)	270	(29%)	136	(25%)	27	(27%)	57	(23%)	52	(27%)
≥ 2	739	(51%)	425	(46%)	314	(58%)	59	(60%)	152	(61%)	103	(54%)
Missing	15	(1%)	15	(2%)	0	(0%)	0	(0%)	0	(0%)	0	(0%)
Smoking status (at baseline)												
Never	225	(15%)	137	(15%)	88	(16%)	19	(19%)	40	(16%)	29	(15%)
Former	983	(67%)	633	(69%)	350	(65%)	63	(64%)	155	(62%)	132	(69%)
Current	252	(17%)	149	(16%)	103	(19%)	17	(17%)	55	(22%)	31	(16%)
Missing	3	(0%)	3	(0%)	0	(0%)	0	(0%)	0	(0%)	0	(0%)
*Tumour characteristics*												
Tumour stage												
Ta	403	(28%)	0	(0%)	403	(75%)	99	(100%)	250	(100%)	54	(28%)
T1	907	(62%)	786	(85%)	121	(22%)	0	(0%)	0	(0%)	121	(63%)
Tis	153	(10%)	136	(15%)	17	(3%)	0	(0%)	0	(0%)	17	(9%)
Tumour grade												
1	156	(11%)	24	(3%)	132	(24%)	99	(100%)	29	(12%)	4	(2%)
2	385	(26%)	125	(14%)	260	(48%)	0	(0%)	221	(88%)	39	(22%)
3	894	(61%)	747	(81%)	147	(57%)	0	(0%)	0	(0%)	147	(77%)
Missing	28	(2%)	26	(3%)	2	(0%)	0	(0%)	0	(0%)	2	(1%)
Adjuvant intravesical therapy												
None	447	(31%)	176	(19%)	271	(50%)	91	(92%)	157	(63%)	23	(12%)
Chemotherapy	146	(10%)	17	(2%)	129	(24%)	7	(7%)	82	(33%)	40	(21%)
BCG	808	(55%)	690	(75%)	118	(22%)	1	(1%)	7	(3%)	110	(57%)
BCG + Chemotherapy	62	(4%)	39	(4%)	23	(4%)	0	(0%)	4	(2%)	19	(10%)

*BCG* Bacillus Calmette-Guérin

^a^ Based on an adapted version of the Self-administered Comorbidity Questionnaire [22], including 14 diseases (cardiovascular disease, stroke, high blood pressure, asthma, chronic bronchitis or COPD, diabetes, stomach disease, kidney disease, liver disease, anaemia or other blood disease, thyroid disease, depression, arthrosis, backpain and rheumatism)

The percentage of missing responses was low (< 1%) for all non-sex related scales at all measurement moments of the UroLife study (Table 2). For the sex-related scales, the percentage of missing responses varied between 6.9 and 12.8%, with exception of female sexual problems (41.4–50.0% missing responses).

**Table 2:** Missing responses, floor effects and ceiling effects of scales of the EORTC QLQ-NMIBC24 for 541 NMIBC patients participating in UroLife

Scale	T6wk (n = 541)			T3mo (n = 471)			T15mo (n = 423)		
	% Missing	% Floor	% Ceiling	% Missing	% Floor	% Ceiling	% Missing	% Floor	% Ceiling
*Scales*									
Urinary symptoms	0.2	9.6	0	0.4	10.4	0	0.7	17.7	0
Malaise	0	87.1	0.6	0.2	86.8	0.2	0.7	90.8	0.2
Future worries	0.2	14.4	1.8	0.2	18.3	0.8	0.2	24.6	0.5
Bloating and flatulence	0	46.0	0.6	0.2	43.3	0.6	0.2	48.0	0.2
Sexual functioning	7.8	37.3	0.6	7.4	32.3	0.4	10.6	28.4	0.5
Male sexual problems^a^	10.3	38.3	7.5	12.8	37.2	5.7	12.8	30.5	8.2
*Single items*									
Intravesical treatment issues^b^	0.6	74.5	0.4	0.7	76.1	0.7	0.5	84.1	0.5
Sexual intimacy^c^	7.3	62.8	0.9	6.9	70.1	1.3	7.4	70.5	0.5
Risk of contaminating partner	7.7	52.6	3.4	7.8	61.0	1.3	7.4	71.1	2.1
Sexual enjoyment	9.0	17.5	3.8	9.1	14.3	3.0	8.4	20.5	3.2
Female sexual problems^d^	45.7	14.3	0	50.0	36.7	6.7	41.4	44.8	0

^a^For 426 (T6wk)/368 (T3mo)/328 (T15mo) men

^b^For 502 (T6wk)/451 (T3mo)/402 (T15mo) men and women indicating this item was applicable to them

^c^For 234 (T6wk)/231 (T3mo)/219 (T15mo) men and women indicating to be sexually active

^d^For 35 (T6wk)/30 (T3mo)/29 (T15mo) women indicating to be sexually active

At T6wk, only four of the eleven scales had no floor effect (< 15%) (Table 2). The highest floor effects were observed for malaise (87.1%) and intravesical treatment issues (74.5%). Over time, the percentage of patients with the lowest possible scores decreased for sexual functioning and male sexual problems but remained stable or increased for all other scales. At T15mo, floor effects were present for all scales. No ceiling effects were observed at any assessment point. The percentage of missing responses was low for all scales, except for female sexual problems (45.7%) (Table 2).

### Structural validity

Table 3 shows the results of the multitrait scaling analysis. All items had a within-scale correlation of 0.40 or higher and a correlation of 0.40 or lower with other scales, indicating good convergent and discriminant validity, respectively. The model chi-square significance was < 0.0001, CFI was 0.93, RMSEA was 0.06 and SRMR was 0.04 after excluding female sexual function (question answered by N = 21), indicating an acceptable fit. Standardized factor loadings are presented in Table 4.

**Table 3:** Item convergent and discriminant correlations by scale within the EORTC QLQ-NMIBC24 at each follow-up time point for 541 NMIBC patients participating in UroLife (multitrait scaling analysis and internal consistency)

Scale	T6wk (n = 541)			T3mo (n = 471)			T15mo (n = 423)		
	Con^a^	Dis^b^	α^c^	Con^a^	Dis^b^	α ^c^	Con ^a^	Dis^b^	α ^c^
Urinary symptoms	0.53–0.82	− 0.21 to 0.26	0.85	0.51–0.82	− 0.18 to 0.31	0.86	0.45–0.84	− 0.11 to 0.27	0.82
Malaise	0.57–0.97	− 0.02 to 0.26	0.72	0.61–0.97	− 0.15 to 0.29	0.67	0.61–0.98	− 0.05 to 0.20	0.62
Future worries	0.82–0.89	− 0.06 to 0.25	0.90	0.83–0.87	− 0.07 to 0.26	0.89	0.83–0.88	0.00–0.26	0.90
Bloating and flatulence	0.74–0.89	− 0.07 to 0.22	0.59	0.71–0.89	− 0.05 to 0.27	0.57	0.66–0.90	− 0.13 to 0.23	0.51
Sexual functioning	0.91–0.94	− 0.21 to (− 0.02)	0.83	0.92–0.95	− 0.28 to (− 0.05)	0.86	0.91–0.95	− 0.19 to (− 0.04)	0.85
Male sexual problems	0.83–0.92	− 0.21 to 0.25	0.77	0.88–0.91	− 0.28 to 0.31	0.77	0.85–0.91	− 0.19 to 0.27	0.74

*Con* convergent validity, *dis* discriminant validity

^a^Range of item-scale correlations corrected for overlap

^b^Range of correlations between an item and all other scales

^c^Cronbach’s alpha

**Table 4:** Standardised factor loadings for the EORTC QLQ-NMIBC24 for 541 NMIBC patients participating in UroLife (confirmatory factor analysis)

Scales and items	Item #	Item topic	Standardized factor loadings^b^
Urinary symptom	Item 31	Have you had to urinate frequently during the day?	0.682
	Item 32	Have you had to urinate frequently at night?	0.622
	Item 33	When you felt the urge to pass urine, did you have to hurry to get to the toilet?	0.768
	Item 34	Was it difficult for you to get enough sleep, because you needed to get up frequently at night to urinate?	0.593
	Item 35	Have you had difficulty going out of the house, because you needed to be close to a toilet?	0.586
	Item 36	Have you had any unintentional release (leakage) of urine?	0.334
	Item 37	Have you had pain or a burning feeling when urinating?	0.497
Malaise	Item 38	Did you have a fever?	0.159
	Item 39	Did you feel ill or unwell?	0.591
Future worries	Item 41	Did you worry about having repeated bladder treatments (cystoscopies or instillations)?	0.618
	Item 42	Were you worried about your health in the future?	0.692
	Item 43	Did you worry about the results of examinations and tests?	0.700
	Item 44	Did you worry about possible future treatments?	0.736
Bloating and flatulence	Item 45	Did you have a bloated feeling in your abdomen?	0.696
	Item 46	Have you had flatulence or gas?	0.339
Sexual functioning	Item 47	To what extent were you interested in sex?	0.700
	Item 48	To what extent were you sexually active (with or without sexual intercourse)?	0.542
Male sexual problems	Item 49	For men only: Dit you have difficulty gaining or maintaining an erection?	0.870
	Item 50	For men only: Did you have ejaculation problems (e.g. dry ejaculation)?	0.827
Intravesical treatment issues	Item 40	Did you have trouble arranging your life around the repeated bladder treatment appointments (cystoscopies or instillations)?	0.564
Sexual intimacy	Item 51	Have you felt uncomfortable about being sexually intimate?	0.690
Risk of contaminating partner	Item 52	Have you worried that you may contaminate your partner during sexual contact with the bladder treatment you have been receiving?	0.821
Sexual enjoyment	Item 53	To what extent was sex enjoyable for you?	0.941
Female sexual problems^a^	Item 54	For women only: did you have a dry vagina or other problems during intercourse?	

^a^This scale was excluded from the confirmatory factor analysis

^b^All p values were < 0.0001

### Reliability

Internal consistency of the scales at all time points was adequate (Cronbach’s α > 0.70), with the exception of bloating and flatulence (Cronbach’s α 0.51–0.59) and malaise (Cronbach’s α 0.62–0.67) (Table 3). Test–retest reliability was acceptable for six scales (ICC > 0.70) and fair to moderate for three scales (0.38–0.65) (Table 5). Test–retest reliability was the lowest for malaise (0.07).

**Table 5:** Intraclass correlation coefficient of the QLQ-NMIBC24 subscales for 134 NMIBC patients participating in the test–retest analysis of BlaZIB

Scale	Assessment of stability	Number of stable patients^b^	ICC	(95% CI)	SEM^a^
Urinary symptoms	Urinary function	80	0.70	(0.57–0.80)	10.0
Malaise	Total function	78	0.07	(− 0.15 to 0.29)	12.9
Future worries	Total function	60	0.78	(0.66–0.86)	8.4
Bloating and flatulence	Bowel function	56	0.65	(0.46–0.78)	12.4
Sexual functioning	Sexual function	58	0.82	(0.72–0.89)	8.9
Male sexual problems	Sexual function	49	0.74	(0.57–0.84)	18.3
Intravesical treatment issues	Total function	76	0.38	(0.17–0.56)	11.8
Sexual intimacy	Sexual function	22	0.73	(0.46–0.88)	11.4
Risk of contaminating partner	Sexual function	20	0.40	(− 0.04 to 0.71)	15.2
Sexual enjoyment	Sexual function	20	0.71	(0.40–0.87)	14.9
Female sexual problems	Sexual function	0			

*ICC*, intraclass correlation coefficient, *CI* confidence interval, *SEM* standard error of measure

^a^Using the Restricted Maximum Likelihood (REML) approach

^b^Patients who (self-reported) remained the same with respect to the specific assessment of stability (i.e. urinary, bowel, sexual or total function) between the T12mo and T12mo + 2wk questionnaire

### Construct validity

Correlations between the core questionnaire and the NMIBC-module were low (< 0.40) for nearly all scales (Table 6). Only between emotional function (QLQ-C30) and future worries (QLQ-NMIBC24) a moderate, inverse correlation was observed (− 0.57). This indicates that the content of the core questionnaire and the NMIBC-specific module do not overlap excessively.

**Table 6:** Spearman correlations between scales in the QLQ-C30 and QLQ-NMIBC24 for 541 NMIBC patients participating in UroLife (divergent validity)

QLQ-C30 scales	QLQ-NMIBC24 scales					
	Urinary symptoms (–)^a^	Malaise (–)^a^	Future worries (–)^a^	Bloating and flatulence (–)^a^	Sexual function (+)^a^	Male sexual problems (–)^a^
Physical function (+)^a^	− 0.23	− 0.16	− 0.11	− 0.13	0.24	− 0.18
Role function (+)^a^	− 0.33	− 0.27	− 0.22	− 0.23	0.14	− 0.14
Emotional function (+)^a^	− 0.25	− 0.24	− 0.57	− 0.26	0.10	− 0.12
Cognitive function (+)^a^	− 0.15	− 0.20	− 0.21	− 0.24	0.07	− 0.17
Social function (+)^a^	− 0.31	− 0.26	− 0.32	− 0.25	0.12	− 0.14
Global Quality of Life (+)^a^	− 0.38	− 0.33	− 0.37	− 0.25	0.16	− 0.16
Pain (–)^a^	0.35	0.27	0.31	0.25	− 0.13	0.15
Fatigue (–)^a^	0.26	0.28	0.27	0.26	− 0.15	0.12
Nausea and vomiting (–)^a^	0.14	0.36	0.10	0.17	− 0.03	0.03

^a^The signs (–) and (+) indicate whether higher scores on this scale are worse or better, respectively

Comparison of patients’ scores at T6wk according to their NMIBC risk subgroups indicated that patients with high-risk NMIBC reported more urinary symptoms (ES = 0.41), future worries (ES = 0.51), problems with sexual intimacy (ES = 0.41) and risk of contaminating partner (ES = 0.72) than patients with low-risk NMIBC (Table 7).

**Table 7:** Mean scores (± SD) of EORTC QLQ-NMIBC24 subscales at 6 weeks after diagnosis for 541 NMIBC patients participating in UroLife by risk category (known group validity)

	Patient score, mean (SD)			Effect size^a^	
	Low risk (N = 99)	Intermediate risk (N = 250)	High risk (N = 192)	Intermediate vs. low	High vs. low
Urinary symptoms (–)^d^	24.5 (20.8)	26.3 (20.1)	33.1 (21.3)	0.09	0.41^c^
Malaise (–)^d^	2.4 (10.8)	3.7 (13.7)	4.3 (10.4)	0.11	0.18
Future worries (–)^d^	28.4 (20.8)	30.6 (24.1)	40.1 (24.5)	0.10	0.51^c^
Bloating and flatulence (–)^d^	21.6 (23.2)	15.4 (20.5)	17.8 (20.8)	− 0.28^b^	− 0.17
Sexual functioning (+)^d^	23.3 (22.3)	21.5 (22.9)	21.7 (22.4)	− 0.08	− 0.07
Male sexual problems (–)^d^	30.9 (30.1)	26.5 (33.1)	29.1 (33.1)	− 0.14	− 0.07
Intravesical treatment issues (–)^d^	9.9 (17.6)	8.0 (17.9)	12.7 (20.4)	− 0.11	0.15
Sexual intimacy (–)^d^	9.2 (19.3)	12.1 (21.7)	18.2 (24.2)	0.14	0.41^b^
Risk of contaminating partner (–)^d^	9.7 (21.7)	15.5 (22.8)	29.1 (31.3)	0.26	0.72^c^
Sexual enjoyment (+)^d^	47.2 (28.2)	44.3 (29.3)	44.4 (31.2)	− 0.10	− 0.09
Female sexual problems (–)^d^	37.5 (33.0)	33.3 (19.2)	27.8 (25.1)	− 0.16	0.33

^a^Effect size of 0.2 is considered small, 0.5 moderate, and 0.8 large

^b^p ≤ 0.05

^c^p ≤ 0.001

^d^The signs (–) and (+) indicate whether higher scores on this scale are worse or better, respectively

### Responsiveness

At T15mo, all QLQ NMIBC24 scores improved compared to T6wk, except for malaise, bloating and flatulence, and male sexual problems (ES < 0.2) (Table 8).

**Table 8:** Unadjusted mean scores (± SD) of EORTC QLQ-NMIBC24 subscales for 541 NMIBC patients participating in UroLife at three time points after diagnosis and mean differences at 3 months (T3mo) and 15 months (T15mo) compared to 6 weeks (T6wk) after diagnosis (responsiveness)

	Patient score, mean (SD)			Effect size^a^	
	T6wk (N = 541)	T3mo (N = 471)	T15mo (N = 423)	T3mo vs. T6wk	T15mo vs. T6wk
Urinary symptoms (–)^e^	28.4 (21.0)	22.3 (19.3)	17.8 (16.4)	− 0.27^d^	− 0.53^d^
Malaise (–)^e^	3.7 (12.1)	3.4 (10.5)	2.5 (9.5)	− 0.02	− 0.08
Future worries (–)^e^	33.6 (24.1)	29.5 (22.6)	24.7 (21.4)	− 0.17^d^	− 0.38^d^
Bloating and flatulence (–)^e^	17.4 (21.2)	17.1 (20.4)	14.4 (18.0)	0.00	− 0.14^c^
Sexual functioning (+)^e^	21.9 (22.6)	24.4 (22.2)	27.1 (23.2)	0.09^d^	0.23^d^
Male sexual problems (–)^e^	28.1 (32.6)	27.5 (31.2)	33.3 (33.0)	− 0.01	0.12^b^
Intravesical treatment issues (–)^e^	10.0 (18.8)	9.5 (18.7)	6.2 (15.7)	0.02	− 0.23^d^
Sexual intimacy (–)^e^	13.8 (22.4)	10.4 (20.0)	10.1 (19.2)	− 0.20^c^	− 0.29^c^
Risk of contaminating partner (–)^e^	19.3 (27.0)	14.3 (22.7)	10.0 (21.5)	− 0.20^b^	− 0.46^d^
Sexual enjoyment (+)^e^	45.0 (29.7)	42.9 (26.5)	41.9 (29.1)	− 0.12	− 0.20^b^
Female sexual problems (–)^e^	33.3 (25.8)	18.8 (36.5)	12.7 (24.7)	− 0.34	− 1.16^b^

^a^Effect size was calculated using Cohen’s d statistic (mean difference divided by pooled standard deviation) and 0.2 is considered small, 0.5 moderate, and 0.8 large

^b^p ≤ 0.05

^c^p ≤ 0.01

^d^p ≤ 0.001

^e^The signs (–) and (+) indicate whether higher scores on this scale are worse or better, respectively

### Interpretability

Based on the correlations between the anchor question and the scales of the questionnaire (ranging between − 0.11 and 0.28), the anchor question was deemed inadequate and no MIC values were calculated.

## Discussion

When evaluating the psychometric properties of the EORTC-QLQ-NMIBC24 in Dutch patients with NMIBC, we observed good structural validity, reliability (i.e. internal consistency and test–retest reliability), construct validity (i.e. divergent validity and known groups validity) and responsiveness. The number of missing items were low among patients for whom the items were applicable, with exception of female sexual problems. At all measurement points, multiple floor effects were observed and MIC values could not be determined.

Multitrait scaling analysis and Cronbach’s α for internal consistency supported the scale structure of the QLQ-NMIBC24. Only the bloating and flatulence and malaise scales (at follow-up) did not reach the 0.70 cut off for group level use of the items in these scales. Other authors have reported similar results and also found that the bloating and flatulence [2] and especially the malaise scale [2, 5, 6] seems to yield unsatisfactory results. These results suggest that there is heterogeneity of the two items in the malaise scale, i.e. items cannot be grouped into one scale, and a revision of this scale may be needed.

The high number of scales with floor effects we observed at T6wk has also been found by other studies. Park et al*.* observed floor effects in nine scales and Mogensen et al. found floor effects in 20 out of the 24 items of the questionnaire [5, 6]. Malaise, intravesical treatment issues and bloating and flatulence had the highest percentages of lowest possible scores in all studies (range 43.3% up to 90%) [2, 5, 6]. Floor effects at baseline may impose a problem as further decreases in symptoms and function over time, as is the case in our study, cannot be measured. Reviewing the relevance of scales with high floor effects at baseline might improve the usefulness of this questionnaire.

The low correlations between the scales of the QLQ-C30 and the QLQ-NMIBC24 questionnaires indicate good discriminant validity and added value of the QLQ-NMIBC24 to the core questionnaire. We could not confirm the moderate correlation (> 0.40) between malaise and urinary symptoms of the QLQ-NMIBC24 and the scales of the QLQ-C30 as observed in previous studies, but did confirm the moderate correlation between future worries and emotional function [2, 6].

We found that the QLQ-NMIBC24 is able to discriminate between subgroups (i.e., NMIBC risk profile) and to measure change over time. However, the difference found for malaise by NMIBC risk profile was small and non-significant. Other studies have found significant differences in most scales according to physical function (> 90 vs < 90) [2] and Karnofsky performance status [6]. For gender comparisons, only differences in sexual function and sexual enjoyment were observed [2;6]. All studies were able to detect changes in score over time [2, 6].

For the test–retest reliability and interpretability, we used an anchor-question and measurement scale to determine changes in patient’s health over the course of time in line with previous recommendations [12]. However, it might not be suitable to assess changes in malaise (ICC of 0.07) as other, non-bladder cancer related, health issues may affect malaise as well. Furthermore, both malaise, as a consequence of treatment, and intravesical treatment issues are highly dependent on the timing of the questionnaire in the treatment process, which might also explain the rather low test–retest reliability for these scales. In addition, we were not able to calculate MIC values as the correlations between the scores on the anchor-question and change scores were too low. Other factors, such as the different modes of administration in the test–retest analysis (online vs. paper), may have also contributed to these findings. Future research will be necessary to determine MIC values for the NMIBC24.

A limitation of this study is the response rate for both questionnaires, i.e. 50% and 58% for UroLife and BlaZIB, respectively. Although our response rates are as expected for Dutch patients with NMIBC, selective non-response may affect the generalizability of the scores to the entire Dutch patient population with NMIBC. We do, however, not expect selective non-response as participants of the UroLife study were comparable with respect to age, gender and tumour stage to the non-responders (data not shown). Furthermore, selective loss to follow-up might have affected some of our results concerning comparison of outcomes over time (i.e. responsiveness), although analyses based on patients who participated in all three UroLife questionnaires (T6wk, T6mo and T15mo) showed similar results (data not shown). At last, the sparse number of sexual active women in our study (n = 35 at T6wk; n = 0 test–retest analysis) limited the examination of the item female sexual problems; we omitted this item from the CFA and could not assess the ICC. Previous studies investigating the psychometric properties of the QLQ-NMIBC24 have also dealt with a low number of female sexual active participants [2, 5, 6] and often lower than is considered adequate according to the COSMIN study design checklist for patient-reported outcome measures [7]. As a consequence, it is hard to draw solid conclusions about the single item female sexual problems.

## Conclusions

The QLQ-NMIBC24 questionnaire has in general a good structural validity, reliability, construct validity and responsiveness. The reliability of the scales malaise and bloating and flatulence is, however, suboptimal. Relevant change scores for this questionnaire could not be defined due to the low correlation between our anchor question and change scores.

## Supplementary Information

**Additional file 1.** Four questions added to the T12mo + 2wk QLQ-NMIBC24 questionnaire to assess whether symptoms – in terms of urinary, bowel, sexual and total function – had decreased, remained the same or increased compared to the T12mo questionnaire.

## Data Availability

The data are currently not publicly available.

## Abbreviations

BCG: Bacillus Calmette Guerin
BlaZIB: ‘BlaaskankerZorg In Beeld’ (EN: Insight into bladder cancer care)
CFA: Confirmatory factor analysis
CFI: Comparative Fit Index
CMO: Committee for Human Research
COSMIN: COnsensus-based Standards for the selection of health Measurement INstruments
EAU: European Association of Urology
EORTC: European Organisation for Research and Treatment of Cancer
EORTC: QLQ-C30 European Organisation for Research and Treatment of Cancer Quality of life Questionnaire C30
ESs: Effect sizes
fiml: Full Info Max Likelihood
HRQoL: Health-Related Quality of Life
ICC: intraclass correlation
MIC: minimal important change
ML: Maximum Likelihood
Mo: Month
NCR: Netherlands Cancer Registry
NMIBC: Non-Muscle Invasive Bladder Cancer
PALGA: nationwide network and registry of histopathology and cytopathology in the Netherlands
PROFILES: Patient Reported Outcomes Following Initial treatment and Long term Evaluation of Survivorship
QLQ-NMIBC24: Quality of life Questionnaire for Non-Muscle Invasive Bladder Cancer
RMSEA: Root Mean Square Error of Approximation
ROC: Receiver Operating Curve
SRMR: Standardized Root Mean Square Residual
UroLife: Urothelial cell cancer: Lifestyle, prognosis and quality of Life
SEM: standard error of measurement
TURBT: Transurethral Resection
WMO: the (Dutch) Medical Research Involving Human Subjects Act
Wk: week
