# Supplementary material for: Validation and reliability of the Dutch version of the EORTC QLQ-NMIBC24 Questionnaire Module for patients with non-muscle-invasive bladder cancer
Source: J Patient Rep Outcomes. 2021 Sep 20;5:96. doi: 10.1186/s41687-021-00372-4 (PMC8452806; doi:10.1186/s41687-021-00372-4)
Supplement: Supplementary file 1 — Additional file 1. Four questions added to the T12mo + 2wk QLQ-NMIBC24 questionnaire to assess whether symptoms – in terms of urinary, bowel, sexual and total function – had decreased, remained the same or increased compared to the T12mo questionnaire. [file 41687_2021_372_MOESM1_ESM.docx]

**Appendix A**

**Dutch version** (i.e. patient version)

| KLACHTEN NU vs. TWEE WEEK GELEDEN |
| --- |

**English (translated) version**

| SYMPTOMS NOW vs. TWO WEEKS AGO |
| --- |

| 35 | Did your bladder cancer specific symptoms (bladder, bowel and sexual function) change compared to the previous questionnaire (about two weeks ago)? Please answer by coloring the box that best applies to you. | | | | | | | | | | | | | | | | |
| --- | --- | --- | --- | --- | --- | --- | --- | --- | --- | --- | --- | --- | --- | --- | --- | --- | --- |
|  |  | **Less complaints** | | | | **Equal** | | **More**  **complaints** | | | | | | | **N.A.** | |  |
|  |  |  |  |  |  |  | |  |  | |  | |  |  | |  |  |
|  | 1. Bladder function | 0 | | | | 0 | | 0 | | | | | |  | |  |  |
|  |  |  |  |  |  | |  |  |  |  | |  | |  | |  |  |
|  | 1. Bowel function | 0 | | | | 0 | | 0 | | | | | |  | |  |  |
|  |  |  |  |  |  | |  |  |  |  | |  | |  | |  |  |
|  | 1. Sexual function | 0 | | | | 0 | | 0 | | | | | | 0 | |  |  |
|  |  |  |  |  |  | |  |  |  |  | |  | |  | |  |  |
|  | 1. Total of symptoms | 0 | | | | 0 | | 0 | | | | | |  | |  |  |
|  |  |  |  |  |  | |  |  |  |  | |  | |  | |  |  |
